# Automation and validation of micronucleus detection in the 3D EpiDerm™ human reconstructed skin assay and correlation with 2D dose responses

**DOI:** 10.1093/mutage/geu011

**Published:** 2014-03-27

**Authors:** K. E. Chapman, A. D. Thomas, J. W. Wills, S. Pfuhler, S. H. Doak, G. J. S. Jenkins

**Affiliations:** ^1^DNA Damage Research Group, Institute of Life Science, College of Medicine, Swansea University, Singleton Park, Swansea, Wales, SA2 8PP, UK,; ^2^The Procter and Gamble Company, 11810 East Miami River Road, Cincinnati, OH 45252, USA

## Abstract

Recent restrictions on the testing of cosmetic ingredients in animals have resulted in the need to test the genotoxic potential of chemicals exclusively *in vitro* prior to licensing. However, as current *in vitro* tests produce some misleading positive results, sole reliance on such tests could prevent some chemicals with safe or beneficial exposure levels from being marketed. The 3D human reconstructed skin micronucleus (RSMN) assay is a promising new *in vitro* approach designed to assess genotoxicity of dermally applied compounds. The assay utilises a highly differentiated *in vitro* model of the human epidermis. For the first time, we have applied automated micronucleus detection to this assay using MetaSystems Metafer Slide Scanning Platform (Metafer), demonstrating concordance with manual scoring. The RSMN assay’s fixation protocol was found to be compatible with the Metafer, providing a considerably shorter alternative to the recommended Metafer protocol. Lowest observed genotoxic effect levels (LOGELs) were observed for mitomycin-C at 4.8 µg/ml and methyl methanesulfonate (MMS) at 1750 µg/ml when applied topically to the skin surface. In-medium dosing with MMS produced a LOGEL of 20 µg/ml, which was very similar to the topical LOGEL when considering the total mass of MMS added. Comparisons between 3D medium and 2D LOGELs resulted in a 7-fold difference in total mass of MMS applied to each system, suggesting a protective function of the 3D microarchitecture. Interestingly, hydrogen peroxide (H_2_O_2_), a positive clastogen in 2D systems, tested negative in this assay. A non-genotoxic carcinogen, methyl carbamate, produced negative results, as expected. We also demonstrated expression of the DNA repair protein *N*-methylpurine-DNA glycosylase in EpiDerm™. Our preliminary validation here demonstrates that the RSMN assay may be a valuable follow-up to the current *in vitro* test battery, and together with its automation, could contribute to minimising unnecessary *in vivo* tests by reducing *in vitro* misleading positives.

## Introduction

An understanding of the genotoxic effects of chemicals in humans is imperative for the prediction of genotoxic potential resulting from chemical exposure. This is particularly important for chemicals utilised in products and treatments with repeated human exposure, such as cosmetics, pharmaceuticals and food. Further, following the 7th Amendment to the Cosmetics Directive in 2009, use of laboratory animals in cosmetics testing has been banned in the European Union (1). Due to the fact that *in vivo* testing has previously been heavily relied upon to confirm positive results of *in vitro* genotoxicity assays, it is now vital that *in vitro* tests alone can accurately identify human carcinogens (2). However, *in vitro* tests often exaggerate toxic effects when compared with *in vivo* results, possibly due to lack of normal human tissue structure or dosing strategies being unrealistic. Further, some *in vitro* tests have used p53-deficient cell lines, which tend to be over-sensitive for genotoxic endpoints compared to normal human cells, contributing to misleading positives (3). Therefore, improvement of the current *in vitro* test battery is urgently required, to allow chemicals with safe, or even beneficial, human exposure levels to be identified and subsequently utilised in products and treatments (2).

### 3D EpiDerm™ reconstructed human skin models

An alternative to the current genotoxicity test battery is the inclusion of *in vitro* methods that mimic human tissues, such as EpiDerm™ 3D reconstructed human skin models (1). Such models are predicted to better reflect the microarchitecture of human tissues and more accurately recapitulate human metabolism than cell lines. They are also predicted to exhibit near-normal DNA repair and cell cycle control (1,4). It has been demonstrated that 87% of tested xenobiotic metabolising enzymes were expressed consistently between the EpiDerm™ model and human skin, further supporting the relevance to the human condition (5). As skin is an organ frequently exposed directly to chemicals, particularly via dermal application of cosmetics and occupational exposure, the EpiDerm™ models represent one of the most common human chemical exposure routes (1). The stratum corneum of EpiDerm™ provides more relevant exposure conditions for target cells during topical dosing, avoiding the non-physiological concentrations of test chemical that are often used in *in vitro* tests (1,4). Therefore, an advantage of such models is that realistic concentrations of chemicals may be tested, with any associated kinetic effects that may occur as the test article diffuses from the epidermal surface to the basal layer of proliferating keratinocytes (4). Additionally, the reconstructed skin micronucleus (RSMN) assay has been shown to detect some chemicals that require metabolic activation (6). Previous studies utilising this assay have established that it is reproducible between laboratories in the USA and Europe, and also that it correctly classifies chemicals into either genotoxic or non-genotoxic carcinogens (1,2,5). Application of test chemicals to the medium instead of topically may also serve as a basic model of systemic exposure to chemicals. The hair dye ingredient, *p*-phenylenediamine (PPD), has previously been added to medium of EpiDerm™ models by Hu *et al.* (7), who noted cytotoxicity and evidence of PPD metabolites (7). An aim of this study was to expand the database for the RSMN assay in another European laboratory and to explore medium exposure in more depth. To our knowledge, this is the first publication exploring the use of models from MatTek’s Slovakia laboratory.

### Exploring genotoxic dose responses in 3D systems

In recent years, experimental techniques for the detection of genotoxicity have become more sensitive, allowing more accurate detection of genotoxic dose responses. This has led to non-linear dose responses, for example, threshold dose-responses, being described (8,9). The demonstration of non-linear dose responses has led to the suggestion that low doses of a genotoxin may not pose a risk to health, unlike higher doses. Cellular resistance to genetic damage at low doses may be due to homeostatic maintenance by DNA repair (10), insufficient levels of chemical traversing cell membranes (bioavailability) or the genotoxin reacting with molecules within the cytoplasm and surroundings (8). As most human exposures occur at low doses, it is vital that low-dose effects are studied. While genotoxic thresholds have been well characterised in 2D cell culture, they have not yet been fully investigated with 3D approaches. Furthermore, previous studies involving the 3D EpiDerm™ models have been primarily used for evaluating whether chemicals induce a positive response for micronuclei in this assay, rather than for obtaining complete dose responses. The parameters—no observed genotoxic effect level and lowest observed genotoxic effect level (LOGEL)—are currently used to define non-linear dose responses at low doses (9) but require substantial statistical power. Therefore, by applying an automated micronucleus scoring platform, as discussed in the next section, to the RSMN assay, such analyses of the low-dose region can be performed in 3D cultures. Given the aforementioned advantages of the RSMN assay, defining the low-dose region in primary cells with 3D microarchitecture maybe more robust. With recent interest in the effects of low-dose genotoxicity, this is the first study to investigate the effects in the low-dose region (i.e. a low-dose range which is several fold lower than that for toxic effects) in 3D culture. The non-linear parameters will be used as a convenient comparison between 2D and 3D and not to define thresholds for the chemicals used. We have investigated low doses of three direct-acting, model genotoxins that have previously tested positive for genotoxic thresholds *in vitro* in human lymphoblastoid cell lines (9,11).

### Use of the Metafer Slide Scanning Platform with the RSMN assay

While the RSMN assay has successfully detected genotoxicity of some chemicals in 3D EpiDerm™ models in previous studies, the scoring for micronuclei was completed manually. In this study, an automated approach, using the MetaSystems Metafer Slide Scanning Platform (Metafer), was introduced for the first time to detect micronuclei in keratinocytes isolated from 3D EpiDerm™ models. As manual scoring is laborious and partially subjective, use of the Metafer to identify micronucleus induction in 3D EpiDerm™ keratinocytes minimises delays associated with time-consuming manual scoring and reduces subjectivity (12). The automated approach also results in more cells being scored than with manual scoring. Therefore, we investigated the application of the Metafer in the RSMN assay, using the RSMN assay’s currently recommended harvest protocol (13).

The objective of this study was to evaluate the use of the RSMN assay in our laboratory for characterisation of the genotoxicity dose responses for low doses of four chemicals of diverse mechanism of action and to validate the Metafer system for use as a more efficient method for micronucleus scoring in keratinocytes from the RSMN assay.

## Materials and methods

### Chemicals

Chemicals were purchased from Sigma-Aldrich (Gillingham, UK) and stored according to the manufacturer’s instructions.

### EpiDerm™ tissues

Tissues were purchased from MatTek In Vitro Life Science Laboratories (Bratislava, Slovakia) and ordered 3 weeks in advance. All tissues shipped to Swansea were derived from cells from the same donor (Keratinocyte strain: 4F1188), as confirmed by MatTek. There are 24 tissues in one batch, and this work represents data generated from ~28 individual batches and, therefore, shipments. Following overnight shipping, tissues were kept at 4°C until assay commencement, with the time spent at 4°C standardised between batches to ensure that transfer to 37°C was completed at the same time on the day of arrival.

### Assessing growth of EpiDerm™ during the RSMN assay

**Fig. 1.**
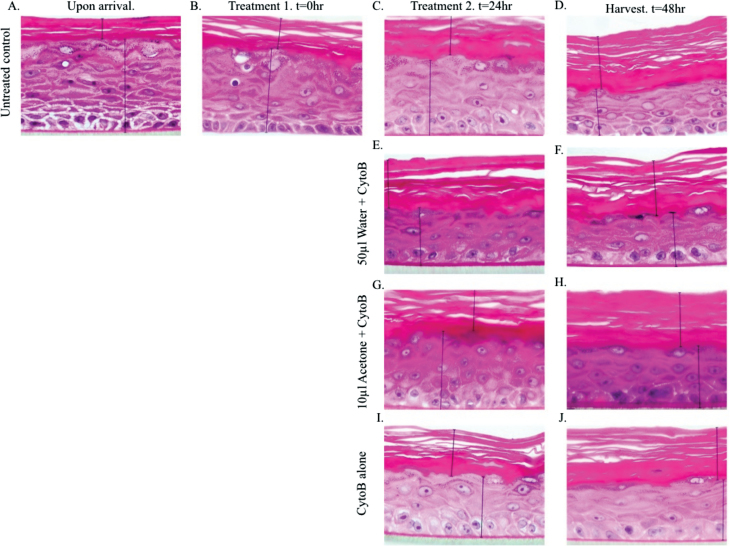
Images of H&E-stained sections documenting the EpiDerm™ skin models for the duration of the assay, during which, the stratum corneum’s thickness increased from 21.8 to 58.3 µm (A−D). No discernable morphological differences were observed after either 24 or 48h following application of water (**E** and **F**) or acetone (**G** and **H**). Only after 48h, binucleated cells were observed (**J**). t = time (h). Vertical lines represent the distance measured for estimation of the thickness of the stratum corneum (top) and the remainder of the tissue model (bottom).

Each day during a typical RSMN assay, EpiDerm™ tissues were sectioned and stained using haematoxylin and eosin (H&E) at Singleton Hospital, Swansea. Sections were analysed using the ×100 objective of an Olympus BX51 light microscope. Size of the stratum corneum was taken as an indicator of cell growth and differentiation (Figure 1).

### RSMN assay

Tissue inserts were inspected for morphological defects, such as blisters. Excess moisture on the surface of the tissue was removed by gentle blotting with sterile gauze. Tissues were placed into 6-well plates with each well containing 900 µl of new maintenance medium (NMM) (MatTek) pre-warmed to 37°C. Tissues were incubated overnight under standard culture conditions (37°C, 5% CO_2_ humidified atmosphere). After 16h, the NMM within the well was aspirated and replaced with 900 µl sterile 37°C NMM containing 3 µg/ml cytochalasin B (cytoB) (Sigma-Aldrich). Test articles were dissolved and diluted in acetone (Fisher Scientific, Loughborough, UK). Ten microlitres of test article was administered onto the topical surface using a positive displacement pipette and the plate rotated for even distribution. Tissues were incubated under standard conditions for 24h. On Day 3, the treatment protocol was repeated and the models incubated for a further 24h with fresh test article.

All tissues were harvested within 48±3h after the first treatment, according to Curren *et al.* (14) and as mentioned previously. For successful trypsin digestion, each tissue was submerged in 5ml Dulbecco’s phosphate-buffered saline (DPBS; Invitrogen, Paisley, UK) for 15min at room temperature (RT) prior to transfer to 5ml ethylenediaminetetraacetic acid (EDTA), pH 8.0 (Ambion, Warrington, UK), diluted to 1mg/ml in DPBS, for 15min at RT. The tissue inserts were blotted before transfer to 1ml 0.25% Trypsin–EDTA pre-warmed to 37°C (Invitrogen). An additional 400 µl of trypsin solution was added directly into the insert and incubated at 37°C for 15min. After this time, tissues were carefully removed from the insert using sterile fine forceps and placed in fresh trypsin solution (1ml). To maximise cell recovery, the insert was rinsed thoroughly and the aforementioned 400 µl was combined with the 1ml in the well. The resulting 1.4ml of the cell suspension was added to 1ml Dulbecco’s modified Eagle’s medium (Invitrogen) (supplemented with foetal bovine serum (10%) (Invitrogen)) to inactivate trypsin.

For in-medium dosing, 10 µl of a stock concentration of methyl methanesulfonate (MMS) was added directly into the medium (890 µl), with the dilution factor accounted for, and mixed. For the mononucleate assay, cytoB was not added to the NMM and tissues were harvested 24h later than for the binucleate assay.

### Cell fixation

Cell suspensions were centrifuged at 100 × *g* for 5min and the supernatant aspirated. One millilitre of potassium chloride (KCl, 75mM; Sigma-Aldrich) pre-warmed to 37°C was added slowly to the pellet. After 3min at RT, 3ml of fixative (3:1 methanol:acetic acid; Fisher Scientific) chilled to 4°C was added. Cells were kept in fixative at 4°C awaiting slide preparation.

### Slide preparation and staining for manual scoring

Cells in fixative were centrifuged at 100 × *g* for 5min at 4°C, the supernatant removed and 5 µl of the cell pellet gently pipetted onto a clean, dry slide as a concentrated dot to minimise cell spreading. Following air drying, slides were stained for 3min using acridine orange (Sigma-Aldrich) diluted to 40 µg/ml in Gurr phosphate buffer (0.1M NaH_4_PO_4_, 0.1M Na_2_HPO_4_) at pH 6.8 (Gibco). Slides were rinsed in Gurr phosphate buffer and left to de-stain for 30min in fresh Gurr phosphate buffer. Micronucleus frequencies were assessed by visualisation under UV light on an Olympus BX50 microscope, using ×100 objective. One thousand binucleated cells were scored for the presence of micronuclei and cell viability assessed by binucleate frequency in 500 cells. Cells with a cytoplasm stained green were excluded from binucleate and micronucleus scores, as these are highly differentiated keratinocytes.

### Slide preparation and staining for automated scoring

For the comparison of automated and manual scoring methodologies, slides for automated scoring were prepared from the same cell pellet used for manual scoring. Following preparation for manual scoring, 200 µl fixative was added and 100 µl of cell suspension was distributed over the entire surface of a wet slide cleaned using 70% ethanol prior to soaking in water. Cell density was adjudged under a light microscope and, if required, the volume of fixative amended accordingly. Once air dried, slides were stored at −20°C awaiting staining. Slides were evenly covered with 20 µl Vectashield Mounting Medium with 4′,6-diamino-2-phenylindole (Vector Laboratories, Peterborough, UK), coverslip applied and kept in the dark for 15min prior to automated scoring using the Metafer system

### Assessment of micronucleus frequency using Metafer

Micronucleus detection in binucleated cells was performed using the parameters specified by A. L. Seager *et al.* (13). As described by Seager *et al.*, parameters for analysis of micronuclei were developed using cell lines. In our studies, Metafer parameters developed for cell lines were unchanged in the analysis of micronuclei in cells from EpiDerm™. Metafer was allowed to systematically scan the slide using the ×10 objective and following scanning, all binucleated cells identified as containing a micronucleus were confirmed by reanalysis of the image by a trained scorer using the ×100 objective. All images of binucleates in the on-screen gallery, which were predicted to not contain micronuclei by Metafer, were also confirmed to be true negatives. Gallery images that appeared to contain a micronucleus were assessed further under ×100 objective (Supplementary Figure 1, available at *Mutagenesis* Online). Therefore, with the need for by-eye confirmation, Metafer analysis could perhaps be considered to currently be a semi-automated scoring approach, rather than fully automated. The criteria for classification of micronuclei, as recommended by Fenech *et al*. (15), were used to characterise micronuclei. Binucleate frequency was scored manually under a Carl Zeiss Axio Imager fluorescence microscope and micronucleus frequency scored using this microscope with the Metafer 4 software, version 3.5 (MetaSystems, Altlussheim, Germany). Separate software for the analysis of frequency of mononucleates, binucleates and multinucleates for cell lines is available for Metafer but was not used in these studies. Approximately 500 cells were scored for binucleate frequency per tissue and 1000 cells were scored for presence of micronuclei. A final micronucleus frequency score was generated based on the number of true micronucleus events following by-eye confirmation. A previously optimised mononucleate classifier was used to detect mononucleates.

### Preparation of positive control

Two 10 µl applications of a 6 µg/ml solution of mitomycin-C (MMC; 0.1875 µg/cm^2^) over 48h were distributed uniformly over the 0.64cm^2^ topical surface area. This gave consistent statistically significant increases in micronucleus frequencies over solvent (negative) controls (2).

### Collaboration with Procter and Gamble Laboratories

To further compare Metafer and manual scoring approaches, Procter and Gamble (P&G), a company that has been involved with validation of the RSMN assay, shipped slides to Swansea University for Metafer analysis. The assay was completed in P&G’s laboratories in the USA and slides were prepared for Metafer analysis using the fixation and slide preparation protocol described previously. Slides were shipped coded to Swansea University, stained and analysed using Metafer, as outlined previously. Comparative acridine orange (manual) scoring was completed in P&G’s laboratories.

### DNA repair gene expression analysis

Following the second chemical treatment, cells were harvested as previously outlined, for tissues treated topically with 0, 1000 and 1900 µg/ml MMS. Following centrifugation at 100 × *g*, supernatant was removed and total RNA extracted from the pellet using the RNeasy Kit (Qiagen, Crawley, UK) and RNase-free DNase Set (Qiagen). Eluted RNA solution was quantified using the NanoDrop (ND 1000) Spectrophotometer, software version 3.1.2. Synthesis of complementary DNA (cDNA) was performed via reverse transcription of 1 µg RNA using the Quantitect Reverse Transcription Kit (Qiagen) and BioRad T100 Thermal Cycler using conditions specified by the manufacturer. The polymerase chain reaction (PCR) was performed for each cDNA sample, using the GoTaq® Flexi DNA Polymerase kit (Promega, Southampton, UK). *N*-methylpurine-DNA glycosylase (MPG) primers (Sigma-Aldrich): Forward primer: GGTCCGAGTCCCACGAAGCC. Reverse primer: CTGCATGACCTGGGCCCCG. β–actin (housekeeping gene): Forward primer: GATGGCCACGGCTGCTTC. Reverse primer: TGCCTCAGGGCAGCGGAA. Polyacrylamide gels (5.4%) were cast and DNA polyacrylamide gel electrophoresis (PAGE) performed at 170V for 30min. DNA was stained by washing the gel for 7min in 1g/l silver nitrate solution (Sigma-Aldrich), followed by a 3min wash in sodium hydroxide/formaldehyde solution (Sigma-Aldrich) until the bands were visible, followed by submersion in ddH_2_O.

### Micronucleus detection in a human lymphoblastoid cell line

TK6 cells were seeded at a density of 1×10^5^ cells/ml in RPMI 1640 medium (Gibco, Paisley, UK) [supplemented with 10% horse serum (Gibco) and 1% glutamine (Gibco)]. After 24h incubation at 37°C and 5% CO_2_, cells were dosed for 24h with MMS diluted in H_2_O. Following the dosing period, medium was replaced with fresh medium and a further 24h recovery period was allowed. Samples were then harvested using the standard Metafer harvest protocol, as described by A. L. Seager *et al.* (13). A mononucleate classifier was used to detect micronuclei in mononucleates and determine micronucleus frequency, using the aforementioned Metafer protocol.

### Statistical analysis

A two-tailed Fisher’s exact test was used to assess whether statistically significant (*P* ≤ 0.05) differences exist between control and treated sample micronucleus frequencies and to predict LOGELs. Doses demonstrating a statistically significant increase in micronuclei above control levels are indicated on graphs by an asterisk. All error bars represent standard deviation around the mean of three biological replicates. Pearson’s product moment correlation was used to determine whether a correlation exists between two separate treatments, where a correlation coefficient, *r*, of +1 is a total positive correlation, 0 is no correlation and −1 is a total negative correlation. The Broken Stick Model (16) was used to predict whether the data fitted a threshold or linear dose response for genotoxicity. Unless otherwise stated in figure legends, each data point is the mean (±SD) of three tissues, with each, where possible, from a separate shipment of Epiderm™ models, thus representing three biological replicates in cases where *n* = 3.

## Results

Using three clastogens with different mechanisms of action (MMC, MMS and H_2_O_2_), for which there is a wealth of *in vitro* micronucleus data available, we evaluated the practicality of the RSMN assay as an advancement in traditional cell culture techniques for modelling genotoxicity and constructing dose-responses. A non-genotoxic carcinogen, methyl carbamate, was also included to validate the specificity of the assay and use of the Metafer Slide Scanning Platform. Dose ranges were selected using preliminary dose-finding studies with escalating doses applied until 50% toxicity (TD_50_) was reached, based on binucleate frequency values.

### Morphology of Epiderm™ models

Sectioning and visualisation of these models was simple, effective and could prove useful in examining the absorption kinetics through skin by similar approaches. We established the morphological changes over time of untreated tissues and those treated with acetone, the recommended solvent, and water, our preferred solvent (Figure 1). No discernable differences were observed between the treatments but, crucially, water gave much lower binucleate frequencies than untreated and acetone-treated tissues (data not shown). Therefore, despite our initial reservations, acetone appears to be the most applicable solvent.

From our experiences with the assay, we were able to observe how the tissues responded to various conditions. For example, delivery of the models was at 4°C overnight from Bratislava and the manufacturers state that tissues are stable for 6 days at this temperature. On one occasion, tissues were delayed by 24h but were without observable defects. Although the vital statistics are within the expected range for a working assay (1,14), several batches were compromised, presumably during transit, and had poor cell yield. Such tissues had a distinct pink coloration once the topical surface had been blotted dry of residual medium following unpacking. It seems likely that the observed pink colouration was due to a leak in the polymer at the bottom of each tissue support, leading to movement of media into the tissue. Remnants of dried medium remained on the topical surface, giving the appearance of a red ring around the perimeter of some tissues, which could not be removed by sterile gauze. However, this had no adverse effect on tissue behaviour. Incidentally, blotting the topical surface to remove condensation accumulated during transit was important for ensuring optimal development of the models. This step was omitted on one occasion and this appeared to reduce binucleate frequency.

### Reproducibility

**Fig. 2.**
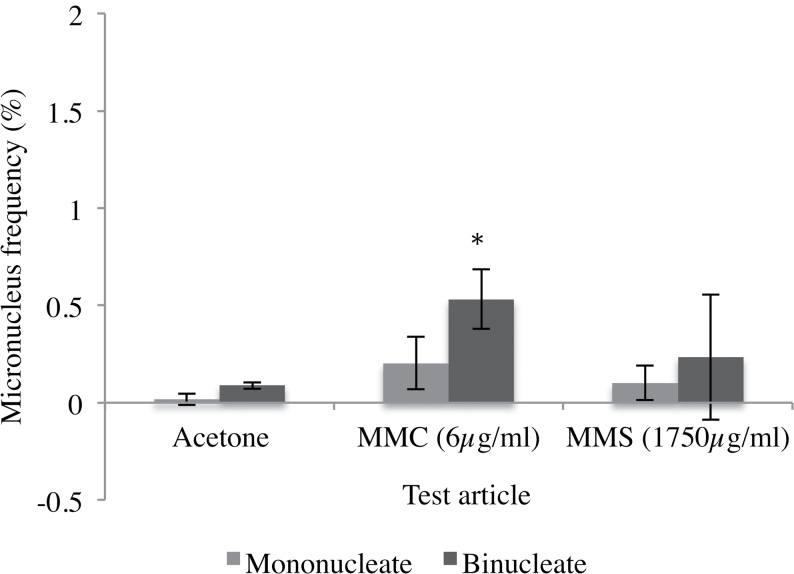
The frequency of mononucleates and binucleates containing a micronucleus (%), according to the mononucleate assay (no cytoB added) and corresponding binucleate assay tissues (*n* = 3), respectively. Approximately 2000 cells were scored per replicate for the mononucleate assay using Metafer. Two 10 µl topical applications of 6 µg/ml (0.1875 µg/cm^2^) MMC or 1750 µg/ml (54.6875 µg/cm^2^) MMS were administered over 48h. A statistically significant difference was observed between mononucleate and binucleate micronucleus frequency for MMC (*P* = 0.02), while acetone (*P* = 0.20) and MMS 1750 µg/ml (*P* = 0.07) did not demonstrate a significant difference between the two assays. Binucleate frequencies for samples with cytoB added: acetone, 58.02±2.36%; MMC, 47.34±2.43%; MMS, 49.29±4.76%. *Represents a *P* ≤ 0.05 relative to the mononucleate assay.

Low inter-batch variability (where a batch is defined as an individual plate of 24 tissues; each batch was usually shipped independently, unless two batches were ordered to arrive in one package) was generally observed in tissues processed in our laboratory, suggesting that the assay is reproducible and supporting previous findings (2). It is also worth noting that as the same donors are used for tissues cultured in both MatTek’s USA and Slovakia laboratories (S. Letasiová, MatTek, personal communication), direct comparisons between the two MatTek laboratories is a possibility. Historical solvent control data from our laboratory were concordant with levels of previous studies (1,2), with a micronucleus frequency of 0.05% (±0.05%, *n* = 26) (Figure 2). Historical binucleate frequency for the solvent control was 52.9±7.75%, which also corresponded with previous studies and was within the expected range (2,17).

**Fig. 3.**
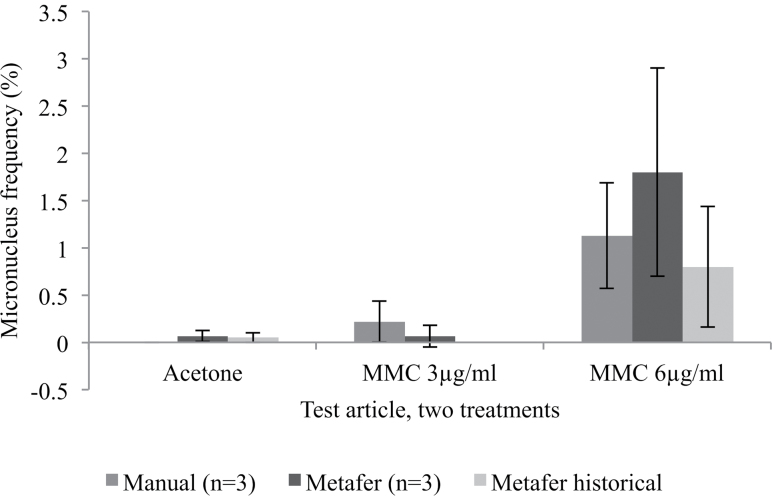
Comparison of frequency of micronucleus events in binucleated cells obtained via manual and automated methods of scoring (*n* = 3). Pearson’s product moment correlation indicated a very strong positive correlation between the manual and automated scores for 6 µg/ml MMC treatments (*r* = 0.921). A strong positive correlation was obtained for 3 µg/ml MMC (*r* = 0.520). No statistically significant difference was observed between manual and automated scores for the solvent (acetone) control. Historical micronucleus frequencies for Metafer for solvent control (26 tissues) and 6 µg/ml MMC (18 tissues) are also included.

MMC positive control data were also reproducible (0.8±0.64%) (*n* = 18) (Figure 3). Although caution is urged with regard to large inter-batch variation received over an 18-week period, our positive control data remain significantly greater than solvent control (Figure 3). Previous inter-laboratory studies have demonstrated variation in micronucleus induction at 6 µg/ml MMC, with frequencies <0.7% reported which are still significantly greater than solvent control levels (2). Further, our historical positive control micronucleus frequency is also around 14× greater than the solvent control frequency, yet it is clear that the aforementioned overlap is an observation that would need addressing for regulatory acceptance. Interestingly, the historical positive control binucleate frequency of 44.2±9.04% is remarkably similar to that observed in three USA-based laboratories, which ranged from 40.9 to 47.1% (2). Overall cell yield was also within the ranges achieved in previous studies (2), according to trypan blue-based cell counting from early batches, which generally yielded between 180000 and 345000 cells.

LOGELs for topical MMS treatments in other laboratories ranged between 600 and 1000 µg/ml (1,2). Considering the inevitable, if slight, variation between laboratories performing the assay, and distributors and donors of the models (2), these figures are within around 3× of our LOGEL for topical MMS of 1750 µg/ml, a relatively minor variation. As mentioned previously, variability was also observed for binucleate frequency at high doses of H_2_O_2_, yet micronucleus frequency remained at background levels.

### The mononucleate assay is less sensitive than the binucleate assay for detection of micronuclei

To validate the influence of cytokinesis block for the RSMN assay’s sub-acute dosing approach (two separate doses of cytoB and test chemical, rather than a single, acute dose, as per 2D), the mononucleate assay was performed (Figure 2). Micronucleus frequency for the binucleate assay in 3D tissues was greater than for the mononucleate assay for the three test articles investigated: there was a 2.9-fold difference for acetone, 3.4-fold for 6 µg/ml MMC positive control and 2.3-fold for MMS treatment. However, only MMC gave a statistically significant difference between mononucleate and binucleate frequency.

### Applicability of the Metafer for detecting micronucleus events in keratinocytes

Tissues were treated with a solvent control and 6 µg/ml MMC per day for 2 days to elicit a positive response. From the same tissue, slides were prepared and stained for manual and automated scoring methods allowing for a direct comparison (Figure 3). Although Metafer produced a greater micronucleus frequency than manual for MMC, a strong positive correlation (*r* = 0.921) was observed between the two methodologies, indicating their congruency. There was no significant difference between the two methodologies for the solvent control (*P* < 0.05), and although Metafer was again slightly greater, producing 0.07% compared to 0% for manual, the Metafer value fell within the expected range (14). Due to the advantages of automation over manual methods, automated scoring was preferred and hereafter utilised in the detection of micronuclei. We also found that counting samples of cells upon harvesting with trypan blue, as recommended by MatTek In Vitro Life Science Laboratories, did not give reproducible scores for toxicity. Instead, binucleate frequency was used as a toxicity measure, as mentioned previously.

### MMC produced a LOGEL for micronucleus induction when applied topically

**Fig. 4.**
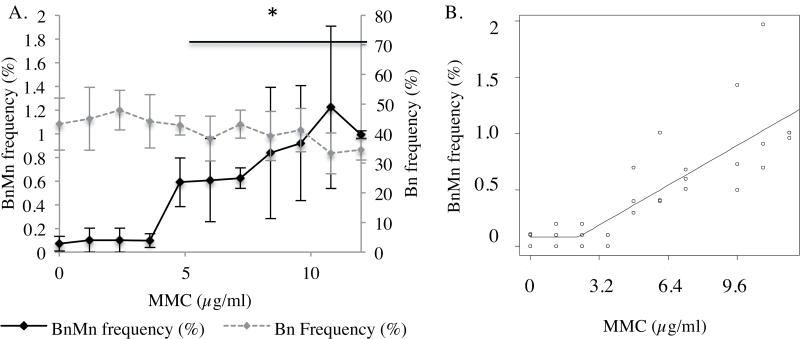
(**A**) Dose response for frequencies of micronuclei and binucleated cells at low doses of MMC. A LOGEL was found at 4.8 µg/ml (*P* = 0.004). Subsequent broken stick modelling (**B**) of these data did not discount a linear response for micronucleus induction (*P* = 0.3). *Represents a *P* ≤ 0.05 relative to the untreated control.

In this assay, 48 µg/ml MMC (a total of 1.5 µg/cm^2^ over 48h) was found to be the TD_50_. The TD_50_ achieved was greater than that reported in the literature, possibly due to differences in miscibility of MMC, although the reason for this is unknown and warrants further investigation (2). The dose–response in Figure 4 was at low doses of MMC, at least 4-fold lower than the TD_50_ to establish low-dose trends. Doses up to 3.6 µg/ml MMC did not cause a significant increase in micronucleus frequency compared to the solvent control. The lowest alpha value was *P* = 0.685 at 1.2 µg/ml MMC. The lowest dose causing significant micronucleus induction over the solvent control was 4.8 µg/ml for topical application of MMC (*P* = 0.03) (Figure 4A), thus representing the LOGEL. However, subsequent broken stick dose–response modelling (16) (Figure 4B), used to define a threshold doses in non-linear dose responses, found no evidence of a threshold and therefore, the null hypothesis of a linear dose–response cannot be rejected (*P* > 0.05). The model may, however, be skewed by the variation at higher doses.

### H_2_O_2_ and methyl carbamate did not cause an increase in micronucleus frequency when applied topically

**Fig. 5.**
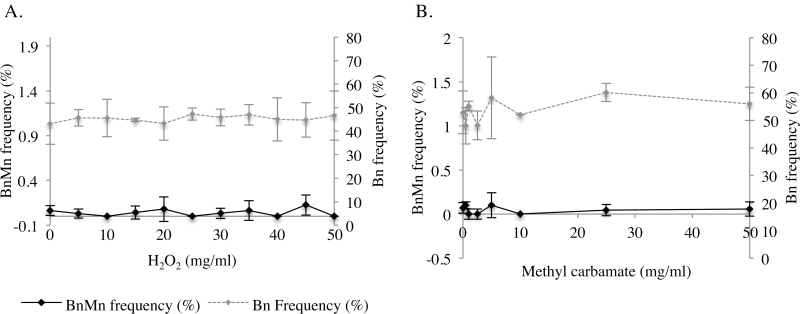
Dose responses for frequencies of micronuclei and binucleated cells (%) for (**A**) H_2_O_2_ (*n* = 3) and (**B**) Methyl carbamate (*n* = 2). No statistically significant differences were observed between the solvent control and treated samples (*P* > 0.05).

Topical administrations of doses of H_2_O_2_ exceeding 35mg/ml (>1.1 µg/cm^2^) caused substantial blistering of the stratum corneum but failed to elicit a positive micronucleus response (Figure 5A). It is interesting to note that other parameters may play a role in determining ‘maximum tolerated doses’ other than the TD_50_ in 3D models. The highest dose of H_2_O_2_ tested for micronucleus induction (50mg/ml) caused a 45% reduction in binucleate frequency compared to control without an increase in micronuclei events. However, there was considerable variation between tissues where binucleate frequency, following treatment with 60mg/ml, which ranged from 45 to 12.5%, thereby giving misleading TD_50_ values, which could impact on hazard identification by these models (3).

In other studies performed within our laboratory, Metafer has consistently given slightly higher micronucleus frequencies than manual scoring. We questioned the specificity of the Metafer and whether this slight over-exaggeration would lead to false positives. Therefore, methyl carbamate, a non-genotoxic carcinogen, was used to validate the specificity of the assay and scoring approach. Methyl carbamate elicited a negative response for micronucleus induction, with no LOGEL (*P* > 0.05) (Figure 5B). Using a non-genotoxin suggests that there is concordance between manual and Metafer scoring, and that the Metafer is not likely to over-predict micronucleus frequency. The doses used here were 100-fold greater than those previously used *in vitro* to conclude that methyl carbamate is non-genotoxic (18). Despite this, toxicity was not observed at these higher doses for methyl carbamate in EpiDerm™ models. Higher doses (up to 800 µg/ml, data not shown) resulted in a reduction of binucleate frequency to 39.1%, without an increase in micronucleus frequency (*n* = 2). As TD_50_ was not observed, the dose range utilised was perhaps not optimal.

### MMS induced LOGELs for both topical and medium dosing

**Fig. 6.**
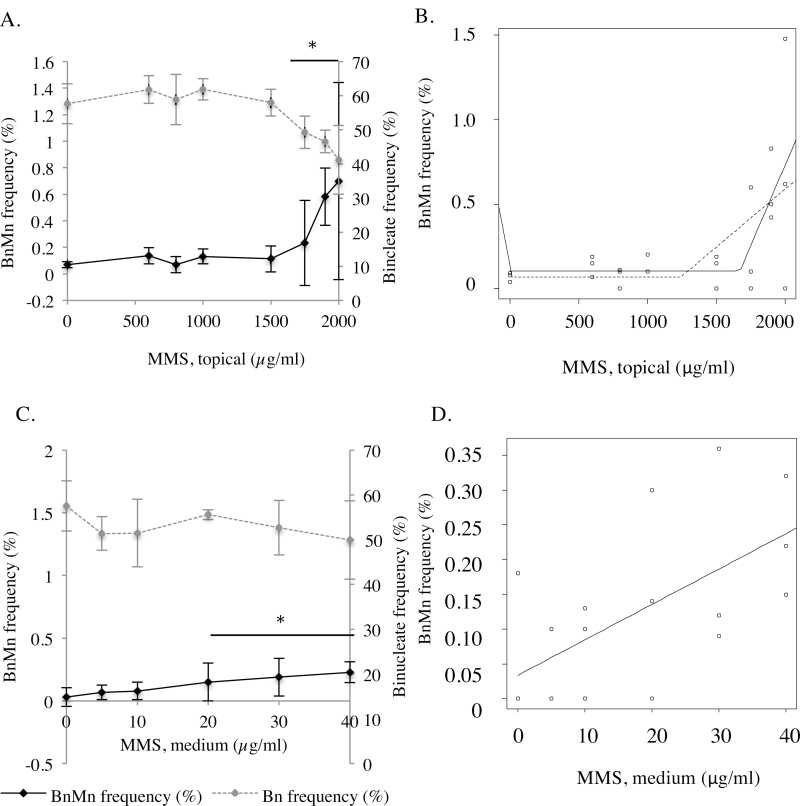
(**A**) Dose–response for frequencies of micronuclei and binucleated cells (%) for MMS applied topically (*n* = 3). LOGEL = 1750 µg/ml (54.69 µg/cm^2^) (*P* = 0.002). (**B**) Broken stick model analysis (16) for data in Figure 6A indicates a non-linear dose–response (*P* = 0.01). (**C**) Dose–response for frequencies of micronuclei and binucleated cells (%) for MMS applied to medium (*n* = 3). LOGEL = 20 µg/ml (0.625 µg/cm^2^) (*P* = 0.047). Binucleate frequency fell to <50% relative population doubling at doses of 50 µg/ml (*n* = 3). (**D**) Broken stick model analysis (16) for data in Figure 6C indicates a linear dose–response (*P* = 1). *Represents a *P* ≤ 0.05 relative to the untreated control.

We explored topical and medium application of the well-characterised methylating agent and clastogen, MMS. Initially, the selected topical dose range was based upon previous studies where increases in micronucleus frequency above solvent control levels were obtained (1,2). When these were found to give negative results in our laboratory, higher doses were tested until binucleate frequency decreased to <50% of control tissues. Topical application with MMS produced a LOGEL at 1750 µg/ml (54.69 µg/cm^2^) (Figure 6A). Addition of MMS directly into the medium produced a LOGEL of 20 µg/ml (Figure 6C). Whereas broken stick modelling suggests a threshold dose–response for topical application (*P* = 0.047) (Figure 6B), the medium dose–response is, interestingly, linear (*P* = 1) (Figure 6D).

### The EpiDerm™ models express MPG mRNA

**Fig. 7.**
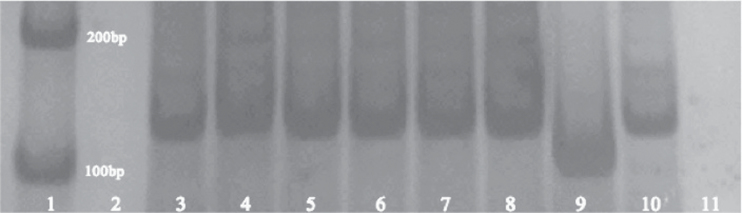
Image of polyacrylamide gel for MPG mRNA expression in the 3D EpiDerm™ models, following two topical applications of MMS (*n* = 2). Lane 1: DNA ladder. Lane 2: empty. Lanes 3 and 4: 10 µl acetone. Lanes 5 and 6: 1000 µg/ml (31.25 µg/cm^2^). Lanes 7 and 8: 1900 µg/ml (59.375 µg/cm^2^). Lane 9: PCR positive control (β-actin). Lane 10: positive control, using RNA from TK6 cells. Lane 11: PCR negative control.

To assess whether 3D tissues express DNA repair genes relevant to the methylating agent used in this study, expression of the glycosylase MPG was measured in RNA extracted from 3D models. In addition, to examine dose-dependent differences in DNA repair enzyme expression following topical treatment of the EpiDerm™ models with MMS, reverse transcription–PCR was performed and visualised using DNA PAGE for MPG. Following treatment, no notable dose-dependent differences were observed for MPG expression (Figure 7). Although not quantitative, levels appeared consistent throughout samples, supporting expression of this DNA repair enzyme in the EpiDerm™ models. Inclusion of a sample of RNA from human lymphoblastoid cell line TK6 for comparison (Lane 10) suggests slightly higher expression of MPG in the EpiDerm™ tissues compared to TK6 cells.

## Discussion

This study further validates the RSMN assay with the EpiDerm™ 3D human skin model and supports its use in the *in vitro* genotoxicity test battery, particularly when assessing cosmetic ingredients. For the first time, we have linked these 3D skin models with automated micronucleus detection using an image analysis system (Metafer). We have used this approach to generate robust dose responses for four chemicals, which have allowed initial comparisons with genotoxicity in traditional 2D cell culture systems.

### Success of inter-laboratory transfer of RSMN methodology

Initial RSMN assays were performed as detailed by Mun *et al*. (1). Accordingly, 3 µg/ml MMC was used to cause a positive response, where micronucleus frequency exceeded 1%. However, in our hands, 3 µg/ml failed to produce such an effect (Figure 3) and 6 µg/ml MMC was used instead. Similarly, a higher dose of MMS was needed (1750 µg/ml) to produce a statistically significant increase in micronucleus frequency over the solvent control compared to previous laboratories’ findings (600 µg/ml) (1), although this was a relatively minor variation, as mentioned previously. The reason for the observed variation is unknown, but this would need to be addressed for standardisation of the RSMN assay.

Furthermore, differences between dose responses for the same chemicals in different laboratories may be due to technical (i.e. chemical preparation and/or dosing techniques), Metafer-based, or donor-related differences within our approach compared to that of previous studies. Such inevitable variations could be due to chemical preparation and dosing technique, for example. Inter-batch variation within a laboratory, as mentioned previously, may also play a role. The lower background micronucleus frequency of EpiDerm™ models compared to cell lines is possibly due to greater genetic stability of primary keratinocytes within the tissues, compared to genetically transformed cell lines, where background micronucleus frequency is potentially more than 10-fold greater (9). This further supports the use of such approaches for providing a more representative model of human exposure *in vitro* than current approaches.

### Automation of micronucleus detection in the RSMN assay

We have demonstrated the Metafer’s potential for automating micronucleus detection in the RSMN assay. We have also found that the assay’s shorter cell fixation protocol is compatible with Metafer analysis and, therefore, with further validation, this protocol might be adapted as a standard harvest protocol for Metafer, reducing time and materials required compared to the current harvest procedure for cell culture experiments. Furthermore, a negative result for methyl carbamate, a non-genotoxic carcinogen, confirmed that Metafer is unlikely to elicit false-positive micronucleus frequency results for non-genotoxic chemicals, despite showing exaggerated micronuclei frequencies compared to manual methods for genotoxins. Given this, we are confident that Metafer analysis is specific for positive genotoxins.

**Fig. 8.**
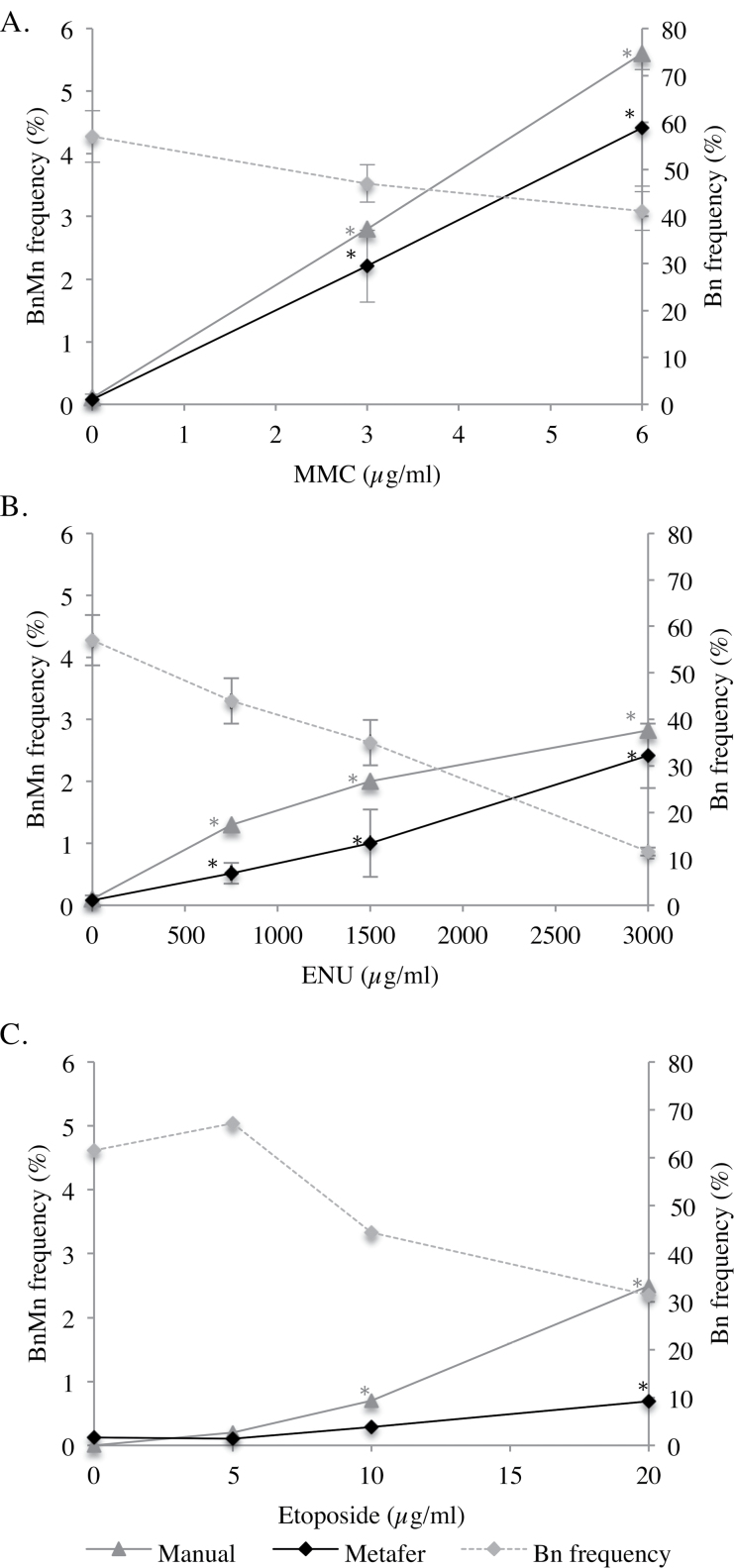
Comparison of micronucleus frequencies using manual scoring, performed at P&G (USA), and Metafer scoring approaches performed at Swansea University. Three chemicals were investigated: (**A**) MMC, (**B**) *N*-ethyl-*N*-nitrosourea (ENU) and (**C**) Etoposide. Both scoring methodologies gave similar outcomes, with asterisks (*) indicating significant increases in micronucleus frequency relative to the solvent control. Corresponding binucleate frequency is also shown.

To further validate the use of Metafer in micronucleus quantitation in the RSMN assay, three coded chemicals, MMC, *N*-ethyl-*N*-nitrosourea and etoposide, were scored using our Metafer System and results compared to manual scoring performed at P&G Laboratories (USA) (Figure 8). In all cases, methodologies showed remarkable similarity in dose-responses. Importantly, this comparison demonstrated that, for the majority, automated scoring correctly distinguished positive results from concurrent controls analogous to manual scoring. Additionally, the inter-study differences we have observed for MMC and MMS cannot be attributed to the scoring methodology. Although Metafer appeared to slightly under-predict micronucleus frequency in general, the only statistically significant difference between the two methodologies occurred at 10 µg/ml etoposide. Here, manual scoring showed this to be significantly different to the solvent control. Upon investigation, it appeared that the magnification used for scanning slides might influence the number of both binucleates and micronuclei detected by the Metafer. For example, use of the ×20 objective appears to enable greater visual clarity and could, therefore, give results closer to manual micronucleus detection than lower magnifications, such as ×10. This may partly be due to the relatively small nuclear diameter of EpiDerm™-derived cells, when compared with that of the lymphoblastoid cell lines that our Metafer system is optimised to detect. Further, P&G uses ×400 magnification to identify micronuclei, while ×100 is used for by-eye confirmation for Metafer; this difference may partly explain the difference observed between Metafer and manual in this case. It would also be possible to, in future, experiment with a lower power objective, more commonly used in manual scoring, with Metafer for by-eye confirmation (e.g. ×60). An interesting observation resulting from this collaboration was that P&G’s 6 µg/ml MMC produced a micronucleus frequency of 4.4% following Metafer analysis (Figure 8), compared to 0.7% at Swansea. This indicates that further investigation may be required, perhaps including inter-laboratory validations. However, as mentioned previously, a highly significant difference was observed between historical solvent and positive control micronucleus frequencies.

Based on the preliminary investigations outlined in this study, it is recommended that further optimisation be completed prior to Metafer being incorporated into this assay routinely. However, these findings are encouraging and provide a solid starting point for combining objective micronucleus scoring with the RSMN assay, giving increased statistical power from analysis of a greater number of cells. Combining 3D skin models with automated micronucleus detection could, therefore, be a major step forward in the quest for animal replacements.

### Correlation with 2D data

**Fig. 9.**
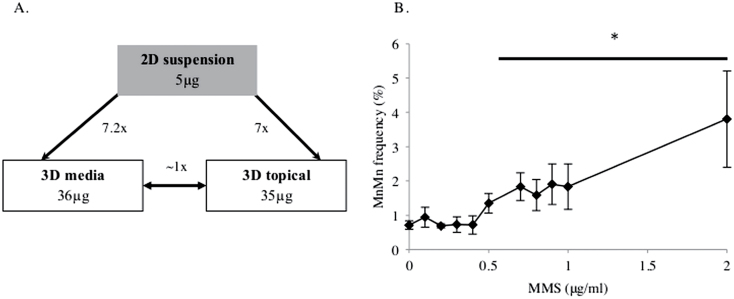
(**A**) Summary of the differences in micronucleus induction in different 2D and 3D cell culture systems in terms of total µg applied to the system. The 2D dose–response referred to is shown in Figure 9B. (**B**) Micronucleus frequency in mononucleate human lymphoblastoid TK6 cells, following 24h treatment with MMS and 24h recovery post-treatment (*n* = 3). A LOGEL was identified at 0.5 µg/ml (*P* < 0.05). *Represents a *P* ≤ 0.05 relative to the untreated control.

The parameters defining non-linearity used here serve as a comparison between 2D and 3D systems and not to define thresholds. The mechanisms of such thresholds are beyond the scope of this study, although differences in LOGELs between 2D and 3D maybe due to assay sensitivity, differential cellular dynamics (cell cycle time), repair capacities and checkpoint proficiencies, or different diffusion and reaction kinetics owing to the different microenvironments. Comparisons between LOGELs obtained in 2D cell culture, such as cells grown in suspension, to those of 3D EpiDerm™ models, may, however, be informative for evaluation of the RSMN assay. For MMS, it is apparent that 2D cell culture models are more susceptible to MMS than the 3D models used here (Figure 6). Around 7×, more chemical was required to elicit a LOGEL in 3D, for both topical and medium dosing, than 2D (Figure 9). Interestingly, approximately the same overall mass elicited a LOGEL in 2D (0.1 µg) and 3D topical (0.096 µg) for MMC (Table I). Whereas topical dosing involves concentrating the chemical over a small area, treatment within a 2D culture disperses chemical over a relatively large volume. This emphasises the difficulty of accurately extrapolating between 2D and 3D topical exposure, due to the contrasting kinetics of the two approaches. A more accurate comparison would be between 2D and 3D medium exposure, as in both cases, the chemical is diluted in medium.

**Table I.** Standardisation of doses of MMC and MMS between 2D and 3D cell culture systems to correct for differences in dose regimes.

	MMC		MMS		
	2D	3D (topical)	2D	3D (topical)	3D (medium)
LOGEL (µg/ml)	0.01	4.8	0.5	1750	20
Final concentration (µg/ml)	0.01	9.6	0.5	3500	40
Total µg applied to system	0.1	0.096	5	35	36

LOGEL for MMC in 2D represents that of L5178Y tk^+/−^ cells (25). LOGEL for 2D was multiplied by 10 to account for 10ml culture, whereas 3D doubled to account for the two doses administered during the RSMN assay. Adjustments for total mass (µg) applied to system due to two 10 µl applications on the topical surface and 900 µl volume of medium.

Interestingly, a similar mass of MMS was required to elicit a LOGEL in both medium (36 µg) and topical (35 µg) treatments in EpiDerm™ (Table I). This perhaps suggests that EpiDerm™’s stratum corneum has limited barrier function, as observed previously (19). This also provides cause to question the suitability of the solvent, which may enhance the permeability of MMS through barrier disruption (20). However, this may also be explained by chemical on the topical surface being highly concentrated in a small area, yet in medium, it is relatively sparse. Therefore, it is necessary to acknowledge that these are extremely different dosing routes and that further investigation with other chemicals is required. With further study, it is possible that ‘sensitivity factors’ could be defined and applied for comparing 2D and 3D, if consistent levels of variation in LOGELs between the two systems are identified.

### Our experience of using the models

H&E stained tissue sections (Figure 1) demonstrated that the stratum corneum thickened over the course of the assay, supporting the models as functional, dynamic representations of the human epidermis. Further, our observations when using the models demonstrated that specific characteristics, such as colour, are important indicators of cell yield and overall health of the models. As mentioned previously, maintaining a dry topical surface is crucial for ensuring that the models remain at their optimum. This became apparent when we questioned the suitability of using the recommended solvent, acetone, by applying a supposedly more biologically compatible alternative, water. When water alone was applied topically, there was a substantial reduction in the binucleate frequency, suggesting that the layer of water remaining on the topical surface prevented cell proliferation and impeded cellular gas exchange. Additionally, a positive dose of 6 µg/ml MMC in acetone was negative in water for micronucleus induction. Whether this is due to water inhibition or acetone potentiation is still unknown. Potential reactivity of acetone with the test article should be realized and may explain the negative result for H_2_O_2_ in Figure 5 (21). Referring to this, a positive result was expected given that H_2_O_2_ has been shown to cause strand breaks in the comet assay in HaCaT transformed keratinocytes (22). Additionally, micronucleus induction following treatment with H_2_O_2_ in human lymphoblastoid cell lines has previously been observed by our research group (10). Whether our negative result is an artefact of the RSMN assay or whether it demonstrates a true null effect in the micronucleus assay through either detoxification (11), efficient oxidative adduct repair or lack of exposure to the deeper epidermal layers is unknown. Indeed, clastogenic effects may be diminished by reaction of H_2_O_2_ with dead cellular matter prior to entry into the cells, and it is possible that chemicals derived from these reactions contributed to reduction in binucleate frequency. Whether there is a full complement of DNA repair and antioxidant capacities or sufficient barrier protection from the stratum corneum remains to be seen. In this instance, medium application of H_2_O_2_ may be informative.

### Mononucleate assay

The mononucleate assay appeared to be less sensitive than the binucleate assay for detection of micronuclei. This may be due to the binucleate assay only considering cells that had divided since the addition of cytoB, whereas the mononucleate assay indiscriminately considers all intact cells (23). Consequently, cells that are senescent, in cell cycle arrest, or pre-apoptotic during the assay, would not have undergone nuclear division since, and indeed due to, chemical exposure (24). Such cells will not contain micronuclei, regardless of the presence of sufficient chromosomal damage (24). This may also occur when the culture conditions do not allow an optimal number of dividing cells for analysis (24). As a result, this is likely to lead to underestimation of micronucleus frequency, generating false-negative results (23). Another disadvantage of the mononucleate assay was that binucleate frequencies could not be obtained for these tissues, due to no cytoB to inhibit cytokinesis.

### Gene expression

Evidence of the mRNA of the DNA repair enzyme, MPG, in tissues of different treatments, indicates that these models are likely to be DNA repair competent. This develops the findings of previous studies of mRNA expression in EpiDerm™, which confirmed comparable expression of xenobiotic metabolising enzymes between human skin and EpiDerm™ (5). However, as presence of mRNA is not necessarily indicative of protein or DNA repair activity, further investigation is required. MPG expression in EpiDerm™ appears slightly greater than for human lymphoblastoid cell line TK6 (Figure 7, Lane 10), suggesting possibly only subtle differences in repair capacity of cell lines and 3D models. Studies of gene expression in EpiDerm™ are particularly important for comparisons with normal human skin, as well as comparisons to existing *in vivo* test models. It was also possible to quantify protein from the models by western blotting of total protein extracts (data not shown).

## Conclusions

We have made advancement in the RSMN assay through inclusion of an automated scoring procedure. Although our data are preliminary, and further validation is required to confirm that Metafer correlates reproducibly with manual scoring, it is clear that there is potential for incorporating the Metafer into this assay. Further optimisation of the ×20 objective in slide scanning by Metafer would be beneficial if the Metafer’s potential in this assay is to be maximised. Combining the assay with the major statistical and practical benefits of Metafer may play a significant role in the reduction of unnecessary animal tests in toxicology. We have also demonstrated that the assay is reproducible and possibly less susceptible to producing misleading positives than existing *in vitro* tests, perhaps providing an alternative follow-up for positives generated by standard *in vitro* genotoxicity assays.

## Supplementary data

Supplementary Figure 1 is available at *Mutagenesis* Online. 

## Funding

This work was supported by the National Centre for the Replacement, Refinement and Reduction of Animals in Research (NC3Rs; Jenkins.G.10-07-2009), the Medical Research Council (MRC; G1000821) and the Engineering and Physical Sciences Research Council (EPSRC; EP/H008683/1).

## Supplementary Material

Supplementary Data
