# Supplementary material for: Automation and validation of micronucleus detection in the 3D EpiDerm™ human reconstructed skin assay and correlation with 2D dose responses
Source: Mutagenesis. 2014 Mar 27;29(3):165–75. doi: 10.1093/mutage/geu011 (PMC3983754; doi:10.1093/mutage/geu011)
Supplement: Supplementary Data [file supp_29_3_165__index.html]

Automation and validation of micronucleus detection in the 3D EpiDerm™ human reconstructed skin assay and correlation with 2D dose responses — Supplementary Data 

# Automation and validation of micronucleus detection in the 3D EpiDerm™ human reconstructed skin assay and correlation with 2D dose responses

## Supplementary Data

Data files

**Files in this Data Supplement:**

- Supplementary Data - Supplementary Data
