# Supplementary material for: Automation and validation of micronucleus detection in the 3D EpiDerm™ human reconstructed skin assay and correlation with 2D dose responses
Source: Mutagenesis. 2014 Mar 27;29(3):165–75. doi: 10.1093/mutage/geu011 (PMC3983754; doi:10.1093/mutage/geu011)
Supplement: Supplementary Data [file supp_geu011_Supplementary_Figures.docx]

**
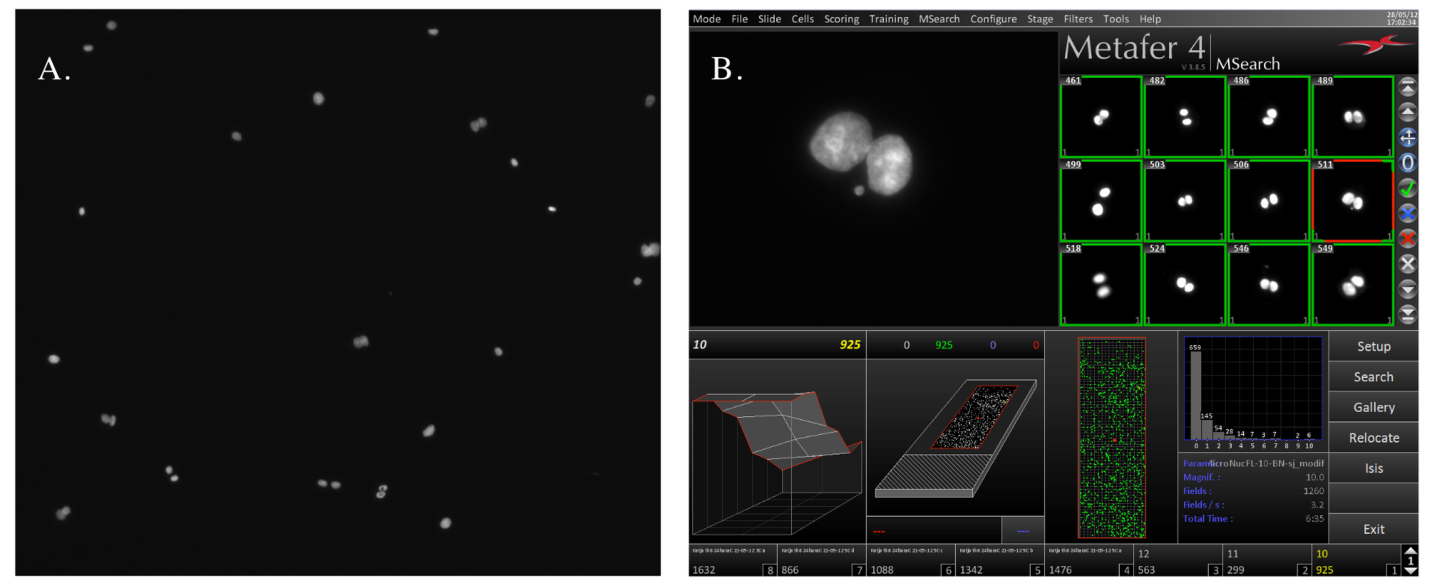
**

**Supplementary Figures**

**Supplementary Fig. 1. A.** Image of the typical cell density on slides prepared for Metafer (viewed using 10x magnification), with DAPI-stained nuclei visible. The relatively low density required for Metafer compared to acridine orange preparation made it possible to distinguish between individual cells, including mononucleates and binucleates, accounting for a lack of visible cytoplasm. **B.** Screenshot from the Metafer 4 software (Version 3.5), including an image of a binucleated EpiDerm^TM^-derived cell containing a micronucleus (100x magnification).
